# Sore Throat; Its Nature, Varieties and Treatment

**Published:** 1880-04

**Authors:** 


*Sore Throat; Its Nature, Varieties and Treatment;* Including the Connections between Affections of the Throat and other Diseases. By Prosser James, M. D., Lecturer on Materia Medica and Therapeutics at the London Hospital, and Physician to Hospital for Diseases of Throat and Chest, Etc., Etc. Publishers: Lindsay & Blackiston, Philadelphia, 1880.

The present publication is the fourth edition of a very useful treatise which is illustrated by handsomely executed hand-colored plates. Commencing with the anatomy of the Throat, all the different varieties of Sore Throat are minutely described with their diagnosis and treatment. Part II relates to different affections, among which are such mouth affections as Apthæ, Thrush, Stomacace, Gangrene of Mouth and Noma. Croup and Diphtheria receive due notice. Part III relates to diseases of Individual Organs, as affections of the Soft Palate and Uvula, also of the Tonsils, Pharynx, Nose, Ears, Æsophagus, Larynx, Voice, Trachea and Bronchi, the whole composing a very useful and carefully prepared work.

